# Update on Anti-EGFR Therapy

**DOI:** 10.1155/2009/157964

**Published:** 2009-09-10

**Authors:** Daniel Chua, Peter Fasching, Brigette Ma, Sumitra Thongprasert, Lori Wirth

**Affiliations:** ^1^Department of Clinical Oncology, Queen Mary Hospital, Pokfulam, Hong Kong; ^2^Department of Gynecologic Oncology, University Hospital Erlangen, 91023 Erlangen, Germany; ^3^Department of Clinical Oncology, Chinese University of Hong Kong, Shatin, Hong Kong; ^4^Department of Medical Oncology, Chiang Mai University, Chiang Mai 50200, Thailand; ^5^Head and Neck Oncology, Dana-Farber Cancer Institute, Harvard Medical School, Boston, MA 02115, USA

Targeted therapy has evolved recently as an important treatment modality for cancer, and the most extensively studied pathways for targeted therapy are those related to epidermal growth factor receptor (EGFR). EGFR is widely expressed in different tumor types and strong expression is associated with higher risk of recurrence/metastasis, poorer survival, and resistance to chemotherapy/radiotherapy. Both monoclonal antibody against EGFR and/or tyrosine kinase inhibitors are now in use clinically for treatment of advanced cancer. With the increasing usage of these agents and the availability of more new agents in the future, knowledge on anti-EGFR therapy is highly relevant to the current state of cancer therapy and to new research directions in the field of oncology.

The focus of this special issue is on the clinical applications of approved or investigational anti-EGFR therapy in solid tumors with emphasis on efficacy, toxicity, response, assessment, multimodality treatment, prognostic factors and predictive markers. The review by A. Harandi et al. provides a comprehensive and updated overview of the frequently used EGFR inhibitors and summarizes clinical efficacy data of these agents and their associated toxicity and management. This review clearly shows the impact of anti-EGFR therapy on cancer treatment since improved treatment results can be seen with the use of anti-EGFR therapy in many common cancers. One of these cancers is colorectal cancer and the approval of anti-EGFR monoclonal antibodies in the treatment of metastatic colorectal cancer has expanded the armamentarium against this disease. H. Loong et al. in their review summarize the historical progress and recent clinical developments of anti-EGFR therapies in the treatment of metastatic CRC and discuss the novel strategies of targeting the EGFR pathway to improve efficacy as well as ongoing research in identifying specific molecular predictors of response.

While the approved use of drugs like the dual kinase inhibitor Lapatinib represents significant advances in the clinical management of breast cancer, confirmatory studies must be considered to foster the use of anti-EGFR therapies including safety, pharmacokinetics, and clinical efficacy in this common cancer. The article by J. Flynn et al. reviews the mechanism of anti-EGFR therapy in breast cancer and summarizes recent advances including the development of improved high-throughput analyses for identifying novel anti-EGFR activity, as well as advances in DNA/RNA-microarray technology for classification purposes, which will contribute to the overall understanding and development of targeted therapy in treatment of breast cancer.

Cancers of the esophagus and stomach present a major health burden worldwide. The impact of cytotoxic agents on the disease has been modest. EGFR pathway has also been implicated in pathophysiology of esophageal and stomach cancers. Recently EGFR inhibitors have been explored in patients with esophageal and gastric cancers, and the results are summarized by T. Dragovich and C. Campen in their review. It also appears that tumors of the distal esophagus and gastroesophageal junction are more sensitive to EGFR blockade than distal gastric adenocarcinoma. 

Similar to cytotoxic agenst, drug resistance remains an important issue and research field in targeted therapy. The article by J. Rolff et al. studied the impact of different resistance markers at protein and mRNA level in patient-derived nonsmall cell lung cancer xenografts in response to erlotinib and other cytotoxic agents. The results suggest that the expression levels of multidrug resistant proteins and mRNA do not play an important role in the drug resistance of nonsmall cell lung cancer. Increased transforming growth factor-*β* (TGF-*β*) expression and EGFR amplification accompany the emergence of highly aggressive human carcinomas, and cooperative signaling between these two growth factor/receptor systems promotes cell migration and synthesis of stromal remodeling factors that in turn regulate tumor invasion, neo-angiogenesis and inflammation. R. Samarakoon et al. studied the role of plasminogen activator inhibitor-1 in carcinogensis and explored its potential role as a novel therapeutic target in combination with EGFR inhibition.

We hope that this special issue will stimulate interests and new researches in the development of anti-EGFR therapy in cancers, particularly the development of personalized medicine based on predictive biomarkers so that therapy can be tailored and optimized in every patient.

*Daniel Chua* *Daniel Chua* *Peter Fasching* *Peter Fasching* *Brigette Ma* *Brigette Ma* *Sumitra Thongprasert* *Sumitra Thongprasert* *Lori Wirth* *Lori Wirth*

